# Promoting enzymatic hydrolysis of lignocellulosic biomass by inexpensive soy protein

**DOI:** 10.1186/s13068-019-1387-x

**Published:** 2019-03-13

**Authors:** Xiaolin Luo, Jing Liu, Peitao Zheng, Meng Li, Yang Zhou, Liulian Huang, Lihui Chen, Li Shuai

**Affiliations:** 1 College of Materials Engineering, Fujian Agriculture and Forestry University, Fuzhou, 350002 China; 2 College of Energy, Xiamen University, Xiamen, 361102 China; 3 Department of Sustainable Biomaterials, Virginia Tech, 230 Cheatham Hall, Blacksburg, VA 24060 USA

**Keywords:** Liquid hot water pretreatment, Enzymatic hydrolysis, Soy protein, Lignocellulosic biomass, Nonproductive binding

## Abstract

**Background:**

Liquid hot water (LHW) pretreatment has been considered as one of the most industrially viable and environment-friendly methods for facilitating the transformation of lignocelluloses into biofuels through biological conversion. However, lignin fragments in pretreatment hydrolysates are preferential to condense with each other and then deposit back onto cellulose surface under severe conditions. Particularly, lignin tends to relocate or redistribute under high-temperature LHW pretreatment conditions. The lignin residues on the cellulose surface would result in significant nonproductive binding of cellulolytic enzymes, and therefore negatively affect the enzymatic conversion (EC) of glucan in pretreated substrates. Although additives such as bovine serum albumin (BSA) and Tween series have been used to reduce nonproductive binding of enzymes through blocking the lignin, the high cost or non-biocompatibility of these additives limits their potential in industrial applications.

**Results:**

Here, we firstly report that a soluble soy protein (SP) extracted from inexpensive defatted soy powder (DSP) showed excellent performance in promoting the EC of glucan in LHW-pretreated lignocellulosic substrates. The addition of the SP (80 mg/g glucan) could readily reduce the cellulase (Celluclast 1.5 L^®^) loading by 8 times from 96.7 to 12.1 mg protein/g glucan and achieve a glucan EC of 80% at a hydrolysis time of 72 h. With the same cellulase (Celluclast 1.5 L^®^) loading (24.2 mg protein/g glucan), the ECs of glucan in LHW-pretreated bamboo, eucalyptus, and Masson pine substrates increased from 57%, 54% and 45% (without SP) to 87%, 94% and 86% (with 80 mg SP/g glucan), respectively. Similar effects were also observed when Cellic CTec2, a newer-generation cellulase preparation, was used. Mechanistic studies indicated that the adsorption of soluble SP onto the surface of lignin residues could reduce the nonproductive binding of cellulolytic enzymes to lignin. The cost of the SP required for effective promotion would be equivalent to the cost of 2.9 mg cellulase (Celluclast 1.5 L^®^) protein (or 1.2 FPU/g glucan), if a proposed semi-simultaneous saccharification and fermentation (semi-SSF) model was used.

**Conclusions:**

Near-complete saccharification of glucan in LHW-pretreated lignocellulosic substrates could be achieved with the addition of the inexpensive and biocompatible SP additive extracted from DSP. This simple but remarkably effective technique could readily contribute to improving the economics of the cellulosic biorefinery industry.

**Electronic supplementary material:**

The online version of this article (10.1186/s13068-019-1387-x) contains supplementary material, which is available to authorized users.

## Background

The development of biorefinery industry requires inexpensive sugar stream for downstream biological and/or chemical conversion [1, 2]. Biomass pretreatment followed by enzymatic hydrolysis has been viewed as a viable way to obtain sugars from biomass due to its fractionation effect and the high selectivity of enzymes for the hydrolysis of polysaccharides (cellulose and hemicellulose) to sugars (e.g., glucose and xylose) [3]. This pathway has been intensively studied for years but two aspects are still in need of improvements to further improve the economics of sugar-based biorefinery processes. First, an environment-friendly pretreatment method is needed. Various pretreatment methods, such as dilute-acid pretreatment [4], alkali pretreatment [5], organosolv pretreatment [e.g. ethanol, tetrahydrofuran, and γ-valerolactone (GVL)] [6, 7] and SPORL (sulfite pretreatment to overcome recalcitrance of lignocellulose) [8], have been successfully developed to produce cellulase-digestible substrates. However, post-treatments are required to remove and/or recover the chemicals or solvents for environmental and economic considerations [8–10]. These additional treatments increase the complexity and the cost of the processing. Second, reducing the use of costly cellulolytic enzymes is still necessary. Klein-Marcuschamer and Liu et al. [11, 12] conducted a techno-economic analysis of bioethanol production from typical lignocelluloses (such as poplar) and demonstrated that the cost of the enzymes would be as high as US $1.47/gal ethanol. Such a high enzyme cost will undoubtedly limit the development of cellulosic biofuel industry.

Liquid hot water pretreatment has been considered as one of the most industrially viable and environment-friendly methods due to several advantages, such as no chemical inputs, no need of recovering or disposing of chemicals or solvents, and no need to separate or wash pretreated slurries prior to enzymatic hydrolysis [13]. Under mild LHW pretreatment conditions, the enzymatic conversions (ECs) of glucan in LHW-pretreated substrates were very low even with a high enzyme loading, mainly because hemicelluloses and lignin were hardly removed [14–17]. Under severe LHW pretreatment conditions, although the significant removal of hemicelluloses and partial removal of lignin could result in digestible substrates, high enzyme loadings (40–60 FPU/g glucan) were required to reach decent ECs of glucan due to nonproductive binding of enzymes to lignin residues [18, 19]. These enzyme loadings were much higher than those (15–20 FPU g/glucan) for enzymatic hydrolysis of substrates pretreated with chemical pretreatment methods such as organosolv pretreatment which produce substrates with low lignin contents [2, 6, 7, 20].

**Fig. 1:**
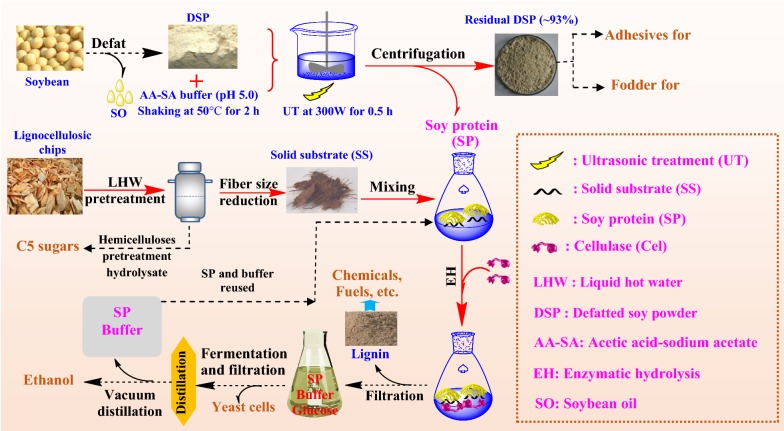
Schematic diagram of the SP-promoted biorefinery process. The processes with solid lines were carried out in this study

To take full advantage of the value of LHW pretreatment, the objective of this work is to reduce the enzyme loadings required for the enzymatic hydrolysis of LHW-pretreated substrates. The use of additives to block lignin and reduce the non-productive binding of enzymes to lignin is the most widely studied way to reduce enzyme loadings [14, 15, 17, 21]. Effective additives include BSA [15], Tween series [21] and so on. However, the high cost or non-biocompatibility of these additives limits their potential in industrial applications. Recently, some researchers found that commercial soy protein (SP) could improve the ECs of glucan in hydrothermally pretreated sugarcane bagasse substrates [22–24]. Unfortunately, Bhagia et al. [25] argued that the cost of the commercial SP required for effective enzymatic hydrolysis promotion was still too high for the cellulosic bioethanol industry. Alternatively, this group tried to use defatted soy powder (DSP) as an additive but the promotion effect was not notable. They ascribed the low promotion effect to the low solubility of DSP in the enzymatic hydrolysis buffer solution. Therefore, we speculated that only soluble SP released from the DSP could effectively interact with the solid lignin residues. Using the enzymatic hydrolysis buffer solution as an extractant, we developed a simple and green method to separate soluble SP directly from inexpensive DSP, which was successfully used to significantly improve the ECs of glucan in LHW-pretreated substrates. The DSP residue after extraction is expected to be utilized for general purposes without any post-treatments (Fig. 1). Experiments were conducted to understand the mechanisms involved in the formation of lignin deposits, the non-productive bindings of enzymes and the promotion effect of SP. We expect that this work can provide a viable and environment-friendly solution for reducing the cost of lignocellulosic biomass saccharification.

## Results and discussion

### Characterization of LHW-pretreated bamboo substrates

To study the effect of pretreatment conditions on the ECs of glucan in LHW-pretreated bamboo substrates, a series of LHW pretreatment experiments were conducted at temperatures of 160–200 °C and reaction times of 20–40 min (Table 1), respectively. Because both the pretreatment time and temperature could affect the severity of a pretreatment process, a severity factor (SF, log*R*_0_) was used to integrate the combinational effect of these two factors according to a previous report [26]. Specifically, any increase in the pretreatment temperature or the pretreatment time could lead to an increase of SF. Table 1 summarized the composition analysis data of the substrates pretreated with different SFs. Overall, when the SF of the LHW pretreatment was intensified from 0 (untreated) to 4.42, the glucan content of the bamboo substrates gradually increased from 39 to 52% (on the basis of o.d. weight of the substrates). During this process, lignin content also gradually increased from 28 to 35% (on the basis of o.d. weight of the substrates), mainly due to the removal of hemicelluloses. With the increase of the SF, up to 87.67% of hemicelluloses in the untreated bamboo was removed while the removals of cellulose and lignin were up to only 14.42% and 20.11%, respectively. These data indicated that LHW pretreatment combining with enzymatic hydrolysis would be a suitable method for fractionating lignocellulosic biomass into three major components, hemicelluloses (or xylose and mannose), cellulose (or glucose), and lignin.

**Table 1:** Compositional analysis of the raw materials (bamboo, eucalyptus, and Masson pine) and corresponding LHW-pretreated substrates

Label^a^	Temp. (°C)^b^	Time (min)	log*R*_0_^b^	SSR^a^ (%)	pH value of hydrolysate	Components content (%)^c^			Components removal (%)^d^		
						Glucan	Xylan	K. lignin	Glucan	Xylan	K. lignin
Untreated B						39.31	23.10	28.17			
Untreated E						46.84	21.73	21.14			
Untreated P						40.37	20.20^e^	28.33			
B-LHW-T160t20	160	20	3.07	91.7	4.41	38.31/41.78	21.73/23.70	26.13/28.49	2.55	6.05	7.29
B-LHW-T160t40	160	40	3.37	90.3	4.18	37.10/41.09	20.46/22.66	25.74/28.50	5.64	11.45	8.70
B-LHW-T180t20	180	20	3.66	84.9	3.91	35.23/41.49	17.18/20.24	25.39/29.90	10.40	25.59	9.91
B-LHW-T180t40	180	40	3.96	73.7	3.64	34.74/47.14	10.76/14.60	23.19/31.47	11.57	53.39	17.63
B-LHW-T200t15	200	15	4.12	69.6	3.62	33.37/47.95	7.63/10.96	22.10/31.75	15.12	66.98	21.58
B-LHW-T200t30	200	30	4.42	64.6	3.26	33.66/52.10	2.85/4.41	22.52/34.86	14.42	87.67	20.11
E-LHW-T200t30	200	30	4.42	65.9	3.08	41.74/63.33	4.28/6.49	17.26/26.19	10.89	80.32	18.37
P-LHW-T200t30	200	30	4.42	69.5	3.31	37.70/54.24	3.46^e^/4.98	23.97/34.49	6.61	82.88^e^	15.38

^a^The numbers after *T* and *t* stand for the pretreatment temperature (°C) and duration (min); B, E, P, SSR and K refer to the abbreviations of bamboo, eucalyptus, Masson pine, solid substrate recovery and Klason, respectively

^b^The “Temp.” represents the abbreviation of temperature; the severity factors (log*R*_0_) for all pretreatments were calculated by the Eq. (3)

^c^The data before and after slashes (/) are the component content based on the o.d. the weight of raw materials and pretreated substrates, respectively

^d^The component removal is calculated by the Eq. (2)

^e^The data represent the sum of the contents of xylan, mannan, and galactan in untreated Masson pine and the LHW-pretreated substrate

Notably, the removal of lignin slightly decreased to 20.11% at an SF of 4.42 after the highest removal of 21.58% was reached at a lower SF of 4.12 (Table 1). This phenomenon was consistent with previous reports [27, 28] and may be ascribed to the deposition of condensed lignin particles and/or pseudo-lignin particles back onto the solid substrate. The high-SF condition (SF, 4.42) could favor the condensation of degraded lignin fragments into those large particles [29, 30] and degradation of carbohydrates to humins (or pseudo-lignin particles) [31–33]. To verify the hypothesis, we measured the size of microparticles in the pretreatment liquors of bamboo immediately after the filtration of the pretreated slurries. We expected that the morphology of the microparticles could provide us with information regarding the formation of lignin and/or pseudo-lignin condensates. We observed the gradual decrease of the microparticle size in the LHW pretreatment liquors with the increasing of SF from 3.07 to 3.96 (Additional file 1: Fig. S1A) and then a jump increase from 3.96 to 4.42 (Additional file 1: Fig. S1A). This phenomenon was very likely caused by the different reaction rates of the two parallel reactions, lignin degradation and lignin condensation [34, 35]. Under low-SF conditions (SFs < 3.96), lignin degradation could be the dominant reaction. In this severity range, lignin particles decreased with the increase of the severity (or the reaction rate) (Additional file 1: Fig S1A). Under high-SF conditions (SFs > 3.96), lignin condensation could become the dominant reaction, leading to the dramatic increase of the microparticle size (Additional file 1: Fig. S1A). The condensation of lignin could be indicated by the increased zeta potentials of lignin microparticles (Additional file 1: Fig. S1B) due to the reduction of negatively charged or polar functional groups (such as carboxyl and aliphatic hydroxyl groups) in condensed lignins [36].

Other than lignin condensation, humins formed from the degradation of carbohydrates could be another factor causing the decreased lignin removal under high-SF conditions [32, 33]. As shown in Additional file 1: Table S1, with the increase of the SF to 4.42, the mass balances of cellulose and hemicelluloses (including furanics) decreased to 83.89% and 65.06%, respectively. The loss of carbohydrates was most likely caused by the degradation of dissolved monosugars to organic acids (such as formic acid, etc.) [19] and insoluble humins (or pseudo-lignin) [32, 33]. In a previous report, the presence of the humins or pseudo-lignin on the surface of pretreated substrates could be confirmed with FTIR spectroscopy by the successful detection of a stretching vibration peak of carbonyl (C=O) in unconjugated ketones at 1720 cm^−1^ [31]. In consistency with this report, we also observed this peak in the FTIR spectra of the bamboo substrates pretreated with SFs higher than 3.96 (Additional file 1: Fig. S2). With the increase of the SF, X-ray photoelectron spectroscopy (XPS) spectra also detected the increase of surface lignin and/or pseudo-lignin coverage (*S*_lig_) from 60% for the bamboo raw material to 86% for the pretreated bamboo substrates (Additional file 1: Fig. S3). According to scanning electron microscopy (SEM) images, more spherical and/or irregular deposits, which were likely lignin and/or pseudo-lignin, were observed on the surface of pretreated bamboo substrates with higher SFs (Additional file 1: Fig. S4). In summary, severe conditions cause not only the re-condensation of degraded lignin but also the formation of humins or pseudo-lignin from carbohydrate degradation. The deposition of these two substances back onto the surface of pretreated substrates causes the observed reduction of lignin removal at the highest-SF condition (SF, 4.42). The effects of the lignin deposits (lignin or pseudo-lignin) on the enzymatic hydrolysis of the pretreated substrates were studied in the following section.

### Enzymatic hydrolysis of LHW-pretreated bamboo substrates

**Fig. 2:**
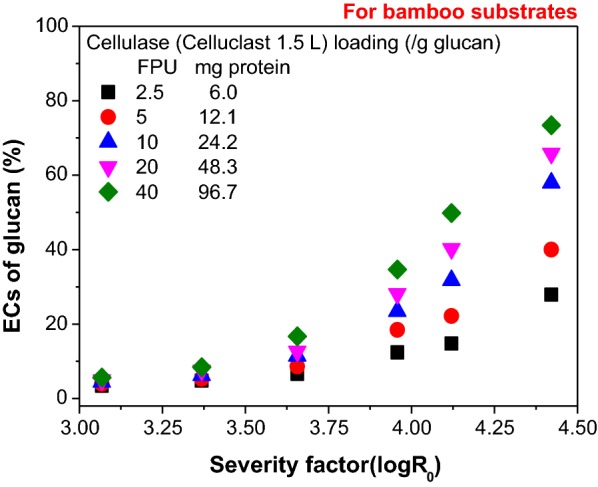
Enzymatic conversions (ECs) of glucan in LHW-pretreated bamboo substrates with various severity factors and different cellulase loadings at a hydrolysis time of 72 h

The ECs of glucan in bamboo substrates pretreated with different SFs were evaluated with different cellulase (Celluclast 1.5 L^®^) loadings. With the increase of the cellulase loading from 6.0 to 96.7 mg protein/g glucan (or 2.5–40 FPU/g glucan), the ECs of glucan increased by 22%, 35% and 46% for the bamboo substrates with SFs of 3.96, 4.12, and 4.42. These results were higher than the ECs increase (2%, 4% and 10%) of substrates with lower SFs of 3.07, 3.37 and 3.66 at a hydrolysis time of 72 h (Fig. 2). This comparison shows that increasing the enzyme loading had a limited positive effect on the ECs of glucan in the low-SF bamboo substrates because of the low digestibility of the substrates. The low digestibility could be caused by two factors: (1) a limited amount of cellulose surface was accessible to enzymes due to the limited removal of hemicellulose and lignin (Table 1) [19, 29, 36] or slight relocation of non-cellulosic components [37], and (2) the cellulose crystalline regions, which have low digestibility, may be hardly destroyed under low-SF conditions [19]. In contrast, the high-SF conditions led to the decent removal of hemicelluloses (Table 1), the redistribution of lignin (Additional file 1: Fig. S4E and F) and possibly the deconstruction of the cellulose crystalline regions [19].

At an increased hydrolysis time of 96 h, no significant changes in the ECs of glucan were observed for low-SF bamboo substrates even with high enzyme loadings (Additional file 1: Table S2), indicating that the enzymatic hydrolysis already ended before 72 h; however, the bamboo enzymatic hydrolysates at 72 h and 96 h still had enzyme activity (Additional file 1: Table S3), suggesting that the ended hydrolysis was mainly caused by the low accessibility or digestibility of the low-SF substrates. This further supports our previous analysis regarding the observation of less EC increments for the low-SF bamboo substrates when the enzyme loading was increased. In contrast, the ECs of glucan for the high-SF substrate (SF, 4.42) continued increasing after 72 h at the enzyme loadings of 48.3 and 96.7 mg protein/g glucan. Particularly, the EC of glucan in this bamboo substrate increased from 73.4% at 72 h to 93.4% at 96 h (Additional file 1: Table S2). The nearly complete hydrolysis indicates that glucan in this bamboo substrate was completely digestible. However, at the low enzyme loading range (6.0–24.2 mg protein/g glucan), the EC of glucan in this substrate (SF, 4.42) hardly changed with the increase of the enzyme loading in this range mostly likely because no effective enzymes remained in the enzymatic hydrolysates after 72 h. This speculation could be supported by the detection of only 3% of the original enzyme activity in the hydrolysate at 72 h (Additional file 1: Table S3). The drop of enzyme activity could be caused by the loss of free enzymes in the enzymatic hydrolysate through nonproductive binding onto lignin or pseudo-lignin deposits (Additional file 1: Figs. S2, S3 and S4F) and/or by the deactivation of enzymes such as the changes of protein conformation at the air–liquid interface [25].

**Fig. 3:**
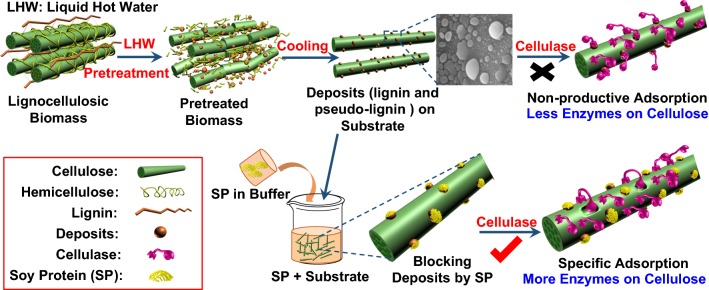
Proposed mechanism of the SP-promoted enzymatic hydrolysis of lignocellulosic substrates

In summary, high-SF LHW pretreatment conditions could generate highly digestible substrates but high enzyme loadings were required to achieve high conversions due to the nonproductive binding of enzymes to lignin and pseudo-lignin deposits (Additional file 1: Fig. S5). The nonproductive binding of enzymes reduced the amount of active enzymes involved in the hydrolysis of glucan [38] and thereby the enzymatic hydrolysis rate of glucan (Fig. 3). Besides, the nonproductive binding of costly enzymes is not economical to the biorefinery industry. Previous and recent studies have suggested that amphiphilic molecules such as Tween 80 [21, 39], lignosulfonate [40], BSA [15], commercial SP [22–24] and DSP [25] can be used to reduce the nonproductive binding of enzymes through blocking the lignin residues on cellulose surface; however, these additives are not industrially viable due to several issues, such as high loadings, high cost, disposal, and the limited promotion effect [25, 41]. To solve these issues, an environment-friendly and inexpensive additive is, thus, needed.

### Extraction of active soy protein from defatted soybean powder

Commercial SP, which is extracted from DSP using chemicals such as acids, alkalis, and solvents in industry, has recently been used as an additive for promoting enzymatic hydrolysis of lignocellulosic biomass [22–24]. However, we found that the SP separated from DSP with the industrial method did not promote the ECs of glucan in the bamboo substrates (Table 2). In industry, the SP is typically separated from DSP by acid or alcohol extraction followed by alkali precipitation and vacuum drying [42]. This process likely causes the denaturation of SP due to the use of acids, alkalis or organic solvents [43]. To avoid the use of these chemicals, acetic acid–sodium acetate (AA–SA) buffer solution (pH 5.0, 50 mmol/L), a widely used buffer solution for enzymatic hydrolysis, was used to extract soluble SP under a mild condition (stirring at 150 rpm and 50 °C for 2 h followed by an ultrasonic treatment at 300 W for 0.5 h) (Fig. 1). The remaining DSP (93%, w/w; Additional file 1: Table S4) could still be used for the production of industrial products such as soy adhesives [44], food additive [45] and feed [46]. The extracted buffer solution containing the soluble SP was directly used for the enzymatic hydrolysis without any post-treatments such as pH adjusting and solvent removal.

**Table 2:** Effect of acid-extracted SP on the ECs of glucan in LHW-pretreated bamboo substrates

mg protein/g glucan		ECs of glucan^b^		
SP^a^ loading	Celluclast 1.5 L^®^ loading	B-LHW-T180t40 (log*R*_0_ 3.96)	B-LHW-T200t15 (log*R*_0_ 4.12)	B-LHW-T200t30 (log*R*_0_ 4.42)
80	12.1	18.1	22.6	40.6
80	24.2	23.4	32.5	57.4

^a^The SP here was separated by acid extraction followed by alkali precipitation and vacuum drying [42]

^b^The time and solid substrate loading of enzymatic hydrolysis were 72 h and 2% (w/v), respectively

### Effect of SP on the ECs of glucan in pretreated substrates

**Fig. 4:**
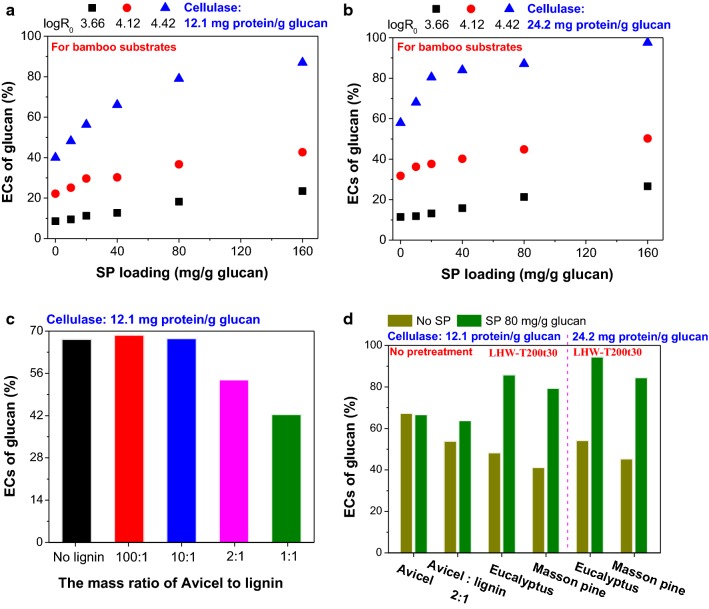
Promotion effects of SP on different lignocellulosic substrates. Effect of SP loadings on the ECs of glucan in substrates with various severity factors at cellulase loadings of **a** 12.1 mg protein (5 FPU)/g glucan and **b** 24.2 mg protein (10 FPU)/g glucan for bamboo; **c** the inhibition of added lignin to the ECs of microcrystalline cellulose (Avicel); **d** the effect of SP addition on Avicel, Avicel–lignin mixture, and eucalyptus and Masson pine substrates. The time of enzymatic hydrolysis was 72 h

To verify the effectiveness of the SP extract, the ECs of glucan in different lignocellulosic substrates (bamboo, eucalyptus, and Masson pine) and in microcrystalline cellulose (Avicel) were investigated with and without SP addition. With the use of the SP extract, different levels of improvements for both enzyme loadings (12.1 and 24.2 mg protein/g glucan) were observed for all substrates pretreated with different SFs (Fig. 4). The results verified our hypothesis that the soluble protein without denaturation could effectively block the surface of lignin or pseudo-lignin deposits via hydrophobic or other interactions [47] and reduce the nonproductive binding of enzymes. The promotion effect of SP became more notable for the bamboo substrates with higher SFs (Fig. 4a, b). For example, when the loading of SP increased from 0 to 160 mg g/glucan, the ECs of glucan in the bamboo substrate pretreated with a SF of 4.42 increased from 40 to 87% at a cellulase (Celluclast 1.5 L^®^) loading of 12.1 mg protein/g glucan and from 58 to 98% at a Celluclast 1.5 L^®^ loading of 24.2 mg protein/g glucan. However, for the bamboo substrate with a SF of 3.66, the ECs only increased from 9 to 23% and 11 to 27%, respectively, with the two different cellulase loadings (Fig. 4a, b). The comparison of the enzymatic hydrolysis of these two substrates further confirmed our analysis that the enzymatic hydrolysis of glucan in the bamboo substrates with low SFs (e.g., 3.66) was mainly limited by the low digestibility of the glucan, while the enzymatic hydrolysis of glucan in the bamboo substrates with high SFs (e.g., 4.42) was mainly affected by the amount of productive enzymes binding to digestible cellulose (or glucan). The effect of nonproductive binding of enzymes to lignin could be further confirmed by studying the hydrolysis of mixtures of Avicel and lignin isolated from the bamboo substrate with an SF of 4.42 (labeled as B-LHW-T200t30). With the increasing addition of the lignin, the EC of Avicel gradually decreased, indicating that enzymes might be increasingly adsorbed by lignin (Fig. 4c). With the addition of the SP extract, the ECs of Avicel–lignin mixture were hardly affected by the addition of the lignin (Fig. 4d), further confirming the promotion effect of SP on the ECs of glucan through blocking lignin or pseudo-lignin deposits. It is noteworthy that SP did not have any promotion effect on the EC of pure Avicel (Fig. 4c), indicating no interaction between enzymes and the non-catalytic SP [25, 47]. We measured the average sizes of enzymes and SP–enzyme mixture in the buffer solution and did not find significant size changes after mixing SP with enzymes (Additional file 1: Table S5).

The success of using SP as an enzymatic hydrolysis promotion additive encouraged us to study its effect on woody biomass, eucalyptus (hardwood) and Masson pine (softwood). After the LHW pretreatment (SF, 4.42) (labeled as E- and P-LHW-T200t30), the addition of SP (80 mg/g glucan) enhanced the ECs of glucan in eucalyptus and Masson pine substrates from 48 to 86% and from 41 to 79%, respectively (Fig. 4d), at a cellulase (Celluclast 1.5 L^®^) loading of 12.1 mg protein/g glucan (5 FPU/g glucan). With the higher cellulase loading (24.2 mg protein or 10 FPU/g glucan) and the addition of SP (80 mg/g glucan), similar increments and higher ECs were observed for eucalyptus (from 54 to 94%) and Masson pine (from 45 to 84%). These results indicate that SP is also an effective additive for promoting the enzymatic hydrolysis of LHW-pretreated woody biomass.

### Comparison of the promotion effect of different additives on enzymatic hydrolysis

**Fig. 5:**
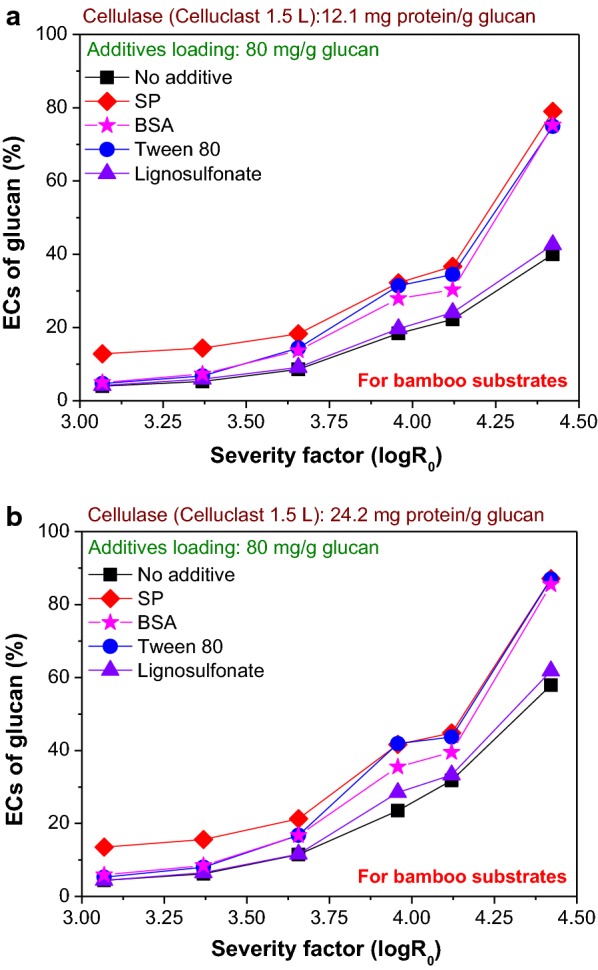
Comparison of the efficiencies of different additives for promoting the ECs of glucan in LHW-pretreated bamboo substrates with various severity factors at cellulase loadings of **a** 12.1 mg protein (5 FPU)/g glucan and **b** 24.2 mg protein (10 FPU)/g glucan at a hydrolysis time of 72 h

The use of other additives including BSA, Tween 80 and lignosulfonate was reported previously. For example, with the additions of BSA (107 mg/g glucan) [15], Tween 80 (385 mg/g glucan) [39] and lignosulfonate (37 mg/g glucan) [40], the ECs of glucan in various pretreated substrates increased by 10% to 24%. To demonstrate the advantage of using the soluble SP extracted from DSP with our method, we compared the promotion effects of these three additives on the ECs of glucan in LHW-pretreated bamboo substrates. The promotion effect of lignosulfonate was not notable (Fig. 5), which was consistent with the previously reported result that only a 10% increase in the EC of glucan was observed [40]. BSA and Tween 80 could achieve a similar effect to SP (Fig. 5), but BSA is extremely expensive [41] and Tween 80 can negatively affect the downstream fermentation processing [48].

**Fig. 6:**
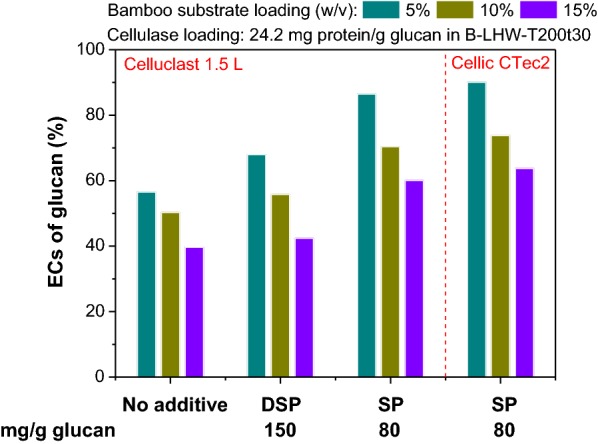
High-consistency ECs of glucan in the LHW-pretreated bamboo substrate labelled as B-LHW-T200t30 without and with additives (DSP 150 mg/g glucan and SP 80 mg/g glucan). Two types of cellulase preparations (Celluclast 1.5 L^®^ and Cellic CTec2, both at a loading of 24.2 mg protein/g glucan) were used for the 72-h enzymatic hydrolysis

Besides, to justify the necessity of separating soluble and active SP from DSP, the promotion effects of DSP, acid-extracted SP, and buffer-extracted SP (Fig. 1) on the EC of glucan in the pretreated bamboo substrate (SF, 4.42) were also compared. Probably due to the insolubility of DSP in the AA–SA buffer solution [25] and the denaturation of acid-extracted SP [43], DSP and acid-extracted SP showed limited promotion effects on the enzymatic hydrolysis (Fig. 6), with the same protein loading (DSP: 150 mg DSP/g glucan, DSP with a crude protein content of 53.4 wt% [49]; SP: 80 mg SP/g glucan). Based on this comparison, we contend that SP extracted with the AA–SA buffer solution could be an ideal renewable additive for improving the ECs of glucan in non-woody and woody biomass pretreated by the environment-friendly LHW pretreatment and possibly the dilute-acid pretreatment.

### SP-promoted high-consistency enzymatic hydrolysis

High-consistency enzymatic hydrolysis is widely considered potential to reduce equipment input and increase sugar and final fermentation ethanol concentration [50, 51]; therefore, we preliminarily investigated the effects of the SP extract on the enzymatic hydrolysis of the LHW-pretreated bamboo substrate (SF, 4.42) at different high substrate loadings. When a cellulase (Celluclast 1.5 L^®^) loading of 24.2 mg protein/g glucan was used, the ECs of glucan in the bamboo substrate without the SP extract were 56.5%, 50.3% and 39.7% for substrate loadings of 5%, 10% and 15% (w/v), respectively. With the addition of the SP (80 mg protein/g glucan), the ECs of glucan in the bamboo substrate were significantly improved (86.4%, 70.4% and 60.1%, respectively) (Fig. 6). For another enzyme preparation (Cellic CTec2), the promotion effects of the SP on the EC of glucan were more notable (90.1%, 73.8% and 63.8%, respectively). The ECs of glucan decreased with the increase of the substrate loading due to the inhibition of accumulated glucose to enzymes [52–55], which was consistent with previous reports from us [56] and another group [57, 58]. This issue could be overcome by simultaneous saccharification and fermentation (SSF) or semi-SSF technologies [56–58]. A systematic study on SP-promoted high-consistency hydrolysis and SSF is ongoing and the results will be reported elsewhere later.

### Cost estimation of SP as an additive during enzymatic hydrolysis

Although many effective additives have been reported, little attention was paid to the loadings and cost of the additives. For some additives, high loadings were required for effective promotion [39], which raises the cost issue. To analyze the industrial viability of SP as an enzymatic hydrolysis additive, we estimated the cost of SP required for effectively promoting enzymatic hydrolysis based on our experimental results.

We speculated that at the end of the enzymatic hydrolysis, only part of SP would be adsorbed by lignin or pseudo-lignin deposits due to the adsorption–desorption equilibrium and the free residual SP in hydrolysates could be reused. For example, to achieve complete hydrolysis of glucan (Fig. 4b) in the bamboo substrate with a SF of 4.42 (labeled as B-LHW-T200t30), a SP loading of 160 mg/g glucan was required when the cellulase (Celluclast 1.5 L^®^) loading was 24.2 mg/g glucan (10 FPU/g glucan). At the end of the hydrolysis, only 28 mg protein/g glucan (Additional file 1: Fig. S5) was adsorbed by the substrate. The reuse of the residual SP could be realized in two ways. One way is to separate SP from the hydrolysate by membrane filtration [59] and another way is to use SSF or semi-SSF technology where the residual SP would be continuously adsorbed by lignins in fresh substrates.

This work has shown that only 28 mg protein/g glucan (Additional file 1: Fig. S5) was adsorbed when 160 mg SP/g glucan and 24 mg enzymes/g glucan (Celluclast 1.5 L^®^, 10 FPU/g glucan) were added. We assumed that 24 mg of enzymes was all adsorbed after the enzymatic hydrolysis, which means at most 4 mg of SP was adsorbed. To continuously conduct the enzymatic hydrolysis of the same amount of the fresh substrate in a semi-SSF process, only 4 mg SP g/glucan along with a fresh enzyme loading of 24 mg/g glucan (Celluclast 1.5 L^®^, 10 FPU/g glucan) (Additional file 1: Table S6) is needed to compensate the loss of the protein caused by lignin and/or pseudo-lignin adsorption. In our semi-SSF model, we assumed that the same amount of the fresh substrate would be fed into a fermenter twenty times, which would result in an average SP loading of 11.6 mg/g glucan. Based on the prices [12] and the loadings of enzymes and commercial SP, the loading of SP (11.6 mg/g glucan) would be equivalent to an enzyme loading of 2.9 mg or 1.2 FPU/g glucan (Details can be referred to the calculation of Additional file 1: Table S6). This estimation suggests that the cost increase resulting from the addition of SP will account for only a small portion of enzymatic hydrolysis cost. It is noteworthy that the cost of SP may be overestimated because our SP extraction process consumes no chemicals and solvents while the commercial SP extraction process does.

## Conclusions

In this work, we made efforts to develop a viable process for conversion of LHW-pretreated lignocellulosic biomass into sugars. A complete enzymatic conversion of glucan to glucose could be achieved with high enzyme loadings. Mechanistic studies indicated that the need for high enzyme loadings was mainly caused by the nonproductive binding of enzymes to lignin and/or pseudo-lignin deposits formed under high-SF pretreatment conditions. To avoid the nonproductive binding of costly enzymes to lignin, we have successfully separated inexpensive SP from DSP using the AA–SA buffer solution as an extractant, which can be used as an additive for the nonproductive binding. The estimation of the cost of SP required for effective enzymatic hydrolysis promotion in a proposed semi-SSF model indicated that the cost of the added SP only accounts for a very small portion of the enzymatic hydrolysis cost. In terms of the effectiveness and convenience, the method could serve as a readily and environment-friendly technique for improving the economics of cellulosic biorefinery after further optimization of the reuse of SP from the perspective of engineering.

## Materials and methods

### Materials

Bamboo and wood (eucalyptus or Masson pine) chips were generously donated by Nanjing Forest Farm (Zhangzhou City, Fujian, China) and Qingshan Paper Co., Ltd. (Sanming City, Fujian, China), respectively. The size of the bamboo and wood chips is around 3.0 × 2.0 × 0.5 cm^3^. Commercial enzymes including cellulases (Celluclast 1.5 L^®^ and Cellic CTec2) and cellobiase (Novozyme 188) and Avicel (Avicel^®^ PH-101) were purchased from Sigma-Aldrich Company (Shanghai, China). According to IUPAC [60] and BCA [61] methods, the activities (FPU/mL) and protein concentration (mg protein/mL) of Celluclast 1.5 L^®^ and Cellic CTec2 were determined to be 51.4 FPU/mL (124.2 mg protein/mL) and 115.3 FPU/mL (174.2 mg protein/mL), respectively. For cellobiase (Novozyme 188), manufacturer-specified activity (413.0 CBU/mL) and protein concentration (218.4 mg protein/mL) were directly used as received. Pierce™ BCA Protein Assay Kit, BSA, lignosulfonate and Tween 80 were ordered from Thermo-Fisher Scientific Life (Rockford, IL, USA) and Tokyo Chemical Industry Co., Ltd. (Shanghai, China), respectively. Defatted soybean powder (DSP) with a crude protein content of 53.4 wt% [49] and an average particle size of 50 μm was provided by Prof. Nairong Chen (FAFU, China). All other reagents and chemicals were of analytical grade and originated from Aladdin^®^ reagent Company (Guangzhou, China).

### LHW pretreatment

LHW pretreatments were conducted in an oil bath digester (YYQ-10-1.25, Jiezhen Sci. & Tech., Nanjing City, China) equipped with ten 1.5-L stainless steel vessels. These stainless steel vessels were fixed on a shelf that mounted in the digester. Glycerol was used as the heating medium of the digester. Prior to the pretreatments, the digester was preheated to a specific temperature with a heating speed of about 3 °C/min. Based on previous studies [14, 17], the temperatures and times of LHW pretreatments ranged from 160 to 200 °C and 20–40 min, respectively. The ratio of water (the moisture content of chips was considered) to chips (on an o.d. basis) for all pretreatments was 6:1 (w/w). Specifically, for a pretreatment trial, 60 g (on an o.d. basis) of bamboo or wood chips (eucalyptus or Masson pine separately) and corresponding deionized water were put into the stainless steel vessel. Once the temperature of the digester reached the desired temperature, the sealed vessel was fixed on the shelf in the digester and rotated at a speed of 4 rpm for the desired time. At the end of the pretreatment, the stainless steel vessel was immediately taken out of the digester and cooled by tap water for about 15 min to quench the reaction. The resultant liquor and solid fraction were separated by vacuum filtration using filter paper on a Büchner funnel. The liquid fraction was collected and stored in a refrigerator (4 °C) for further measurement (Additional file 1: Fig. S1, Table S1). Without size treatment, the pretreated solid fraction still retained the original shape. Part of the wet solid fraction was dried at 121 °C overnight to gravimetrically determine the solid substrate recovery (SSR). Part of the wet solid fraction was directly vacuum dried and then used for SEM and XPS analyses. Vacuum-dried fraction was further ground in a mill (ZM 200, Retsch, Haan, Germany) to pass a screen with a square opening size of 180 μm. The resulted powder was subjected to FTIR (Additional file 1: Fig. S2) and compositional (Table 1) analyses. To eliminate the effect of particle size on subsequent enzymatic hydrolysis, part of the wet solid fraction was initially fibrillated by a laboratory drug mill (XY100, Yongkang Factory, Zhejiang, China) with a motor speed of 22,000 rpm for 20 s and further ground by a micro-plant grinder to particles (1–2 mm). The resulted solid (Fig. 1) was regarded as the pretreated solid substrate for enzymatic hydrolysis and protein adsorption.

The content (*C*, %) of left component (glucan, xylan or Klason lignin) in a pretreated substrate based on the oven dried (o.d.) weight of raw material (untreated bamboo or wood chips) could be calculated as:

1 $$C = \left( {Y \times C_{2} } \right)/100,$$

where *Y* is the solid substrate recovery (%, on the basis of the initial o.d. weight of a raw material); *C*_2_ is the content (%) of component (glucan, xylan or Klason lignin) in a pretreated solid substrate, respectively.

The component removal was calculated as:

2 $$R = 100\left( {C_{1} - C} \right)/C_{1} ,$$

where *R* is the component (glucan, xylan or Klason lignin) removal (%); *C*_1_ is the content (%) of the component (glucan, xylan or Klason lignin) in the o.d. raw material, respectively. The meaning of *C* is same as that shown in Eq. (1).

According to Ref. [26], the combinational effect of pretreatment temperature and time on the severity of LHW pretreatment was integrated by the severity factor (log*R*_0_). Its definition is expressed as follows:

3 $$\log R_{0} = { \log }\left[ {t \times { \exp }\left( {\frac{{T_{\text{i}} - T_{\text{ref}} }}{14.75}} \right)} \right],$$

where *t* is the duration of a pretreatment (min); *T*_i_ is the pretreatment temperature (°C); *T*_ref_ is the base temperature (100 °C).

### SP extraction

DSP (100 g) was added into 500 mL of AA–SA buffer solution (pH 5.0, 50 mmol/L). The mixture was shaken in a speed of 150 rpm at 50 °C for 2 h and then ultrasonically treated at 300 W for 30 min. The resulted slurry was centrifuged (Avanti J-30I, Beckman Coulter Inc., Fullerton, CA, USA) at 5000 rpm for 5 min. The concentrations of SP and background sugar in the supernatant were measured by bicinchoninic acid (BCA) protein assay kit [61] and ion chromatography (IC) [62]. The supernatant containing SP was used directly for subsequent enzymatic hydrolysis. The effects of background concentration of glucose released from DSP to the buffer solution on the calculation of enzymatic hydrolysis efficiency (described in follows) and BCA test (see additional experiment procedures in Additional file 1) were considered.

For comparison, SP was also isolated from DSP by acid extraction followed by alkali precipitation and vacuum drying, according to a reported procedure [42]. The SP obtained by this traditional method (acid-extracted SP) was directly added to the buffer for enzymatic hydrolysis.

### Composition analysis of raw material and substrates

The contents of main components in raw materials (bamboo or another two kinds of wood) and pretreated substrates were measured according to the method developed by National Renewable Energy Laboratory [63]. In general, glucan, xylan and Klason lignin contents were determined by a two-step acid hydrolysis method. First, 0.3 g of the raw biomass powder or the pretreated substrate powder was placed in a pressure-resistant glass vessel containing 3 mL of 72% (w/w) sulfuric acid solution and stirred every 15 min at 30 °C for 1 h. After the treatment, 84 mL of deionized water was added to adjust the final sulfuric acid concentration to 4% (w/w). The vessel was sealed and autoclaved at 121 °C for 1 h. Upon completion, the pressure-resistant vessel was immediately taken out of the autoclave and cooled to room temperature in the air. The solid–liquid mixture was then filtered on a Büchner funnel with filter paper. The filtrate was analyzed by IC (Dionex ICS-5000, Carbopac PA20, US) for the concentrations of five monosaccharides (arabinose, galactose, glucose, xylose, and mannose) [62]. The contents of glucan and xylan in the pretreated solid substrates or raw materials were calculated based on the concentrations of glucose and xylose in the filtrate, respectively. The residual solid was weighed to determine the content of Klason lignin.

### Enzymatic hydrolysis

Enzymatic hydrolysis of LHW-pretreated substrates was conducted with a 2% (w/v) solid loading in an AA–SA buffer (pH 5.0, 50 mmol/L) at 150 rpm and 50 °C for 72 h or 96 h. The loading of Cellulase (Celluclast 1.5 L^®^) ranged from 6.0 to 96.7 mg protein/g glucan (2.5–40 FPU/g glucan) in pretreated substrates and cellobiase loading (mg Novozyme 188 protein/g glucan) was 0.3 times of the cellulase protein loading. To overcome the negative effects of lignin and/or pseudo-lignin deposits on the EC of glucan in the substrates, the additive (i.e., SP extract, BSA, Tween 80 or lignosulfonate) and solid substrate were incubated at 50 °C for 2 h prior to the enzymatic hydrolysis. The required volume of the SP extract for enzymatic hydrolysis was calculated based on the required SP loading (10–160 mg/g glucan) and the SP protein concentration (Additional file 1: Table S4) in the extract.

For high-consistency enzymatic hydrolysis (5–15%, w/v), the enzymatic hydrolysis of LHW-pretreated bamboo substrate (labeled as B-LHW-T200t30) was also conducted in the AA–SA buffer (pH 5.0, 50 mmol/L) at 250 rpm and 50 °C for 72 h. The additive (DSP or SP) was added to the buffer solution prior to the enzymatic hydrolysis. DSP (150 mg, crude protein content: 53.4%) was loaded to maintain the same protein loading as the loading of 80 mg SP protein/g glucan. The pretreated bamboo substrate was separately fed by 5% (w/v) at 0, 24 and 48 h, respectively. After the bamboo substrate was mixed with the additive in the buffer for 2 h, the required enzymes (cellulase and cellobiase) was added (e.g., 2, 26 and 50 h). To test the adaptability of SP to different enzymes, two types of cellulase preparations (Celluclast 1.5 L^®^ and Cellic CTec2) with the same loading (24.2 mg protein/g glucan in substrates) were used for enzymatic hydrolysis experiments. The loading of cellobiase (Novozyme 188) was 7.92 mg protein/g glucan (15 CBU/g glucan) for all high-consistency enzymatic hydrolysis (5–15%, w/v).

In the enzymatic hydrolysis experiment with the highest loadings of additives (DSP or SP) and enzymes, the background concentration of glucose released from DSP and/or enzymes in the buffer solution were detected to be only 0.15 g/L. Therefore, in other enzymatic hydrolysis trials with lower loadings of additives and enzymes, the background glucose concentrations should be lower than 0.15 g/L and therefore were ignored.

A glucose analyzer (2900D, YSI Inc., Yellow Springs, OH, USA) was used to determine the concentration of glucose in the enzymatic hydrolysates [56, 64]. The YSI measurement results correlated well with those of IC (*y* = 1.0381*x *− 0.1166, *R*^2^ = 0.9994) within the glucose concentration range of 0.3–9 g/L. The following equation was used to calculate the ECs of glucan in the substrates.

4 $${\text{EC}} = \frac{{0.9W_{\text{glucose}} }}{{W_{\text{glucan}} }},$$

where *W*_glucose_ is the amount of glucose in the enzymatic hydrolysate (g); *W*_glucan_ is the amount of glucan in the substrates used for enzymatic hydrolysis (on an o.d. basis) (g); 0.9 is the conversion coefficient for glucose to equivalent glucan during enzymatic hydrolysis.

## Additional file

**Additional file 1: Fig. S1.** Effects of severity factors (SFs, logR_0_) on the (A) average size and (B) zeta potential of microparticles in LHW-pretreated bamboo hydrolysates. **Fig. S2.** FTIR spectra of the raw bamboo material and LHW-pretreated bamboo substrates. **Fig. S3.** (A) XPS spectra and (B) S_lig_ of the raw  bamboo material and LHW–pretreated bamboo substrates. **Fig. S4.** SEM images of (A) untreated bamboo, and LHW-pretreated bamboo substrates labeled as (B) B-LHW-T180t20 (SF 3.66), (C) B-LHW-T180t40 (SF 3.96), (D) B-LHW-T200t15 (SF 4.12) and (E) B-LHW-T200t30 (SF 4.42). Fig. S5 Adsorption of cellulase (Celluclast 1.5 L^®^) and SP on the pretreated bamboo substrate (SF 4.42). **Table S1.** Mass balances of carbohydrates in the pretreatment liquors of bamboo. **Table S2.** ECs of glucan in different pretreated bamboo substrates at an enzymatic hydrolysis time of 96 h. **Table S3.** Remaining cellulase (Celluclast 1.5 L^®^) activities (FPU/mL) in the two bamboo enzymatic hydrolysates (SFs of 3.66 and 4.42) after 72 and 96 h hydrolysis at cellulase loadings of 24.2 and 96.7 mg protein/g glucan. **Table S4.** Concentrations of SP and glucose in the AA-SA solution (pH 5, 50 mmol/L) and the corresponding extraction ratio. **Table S5.** Zeta potentials, average sizes, and activities of cellulase, SP and cellulase-SP mixtures. **Table S6.** Cost estimation of SP as an additive during enzymatic hydrolysis.

## Abbreviations

LHW: liquid hot water
SF: severity factor
SP: soy protein
EC: enzymatic conversions
DSP: defatted soybean powder
AA–SA: acetic acid–sodium acetate
BSA: bovine serum albumin
SSF: simultaneous saccharification and fermentation
FPU: filter paper unit
SPORL: sulfite pretreatment to overcome recalcitrance of lignocelluloses
BCA: bicinchoninic acid
