# Supplementary material for: Promoting enzymatic hydrolysis of lignocellulosic biomass by inexpensive soy protein
Source: Biotechnol Biofuels. 2019 Mar 13;12:51. doi: 10.1186/s13068-019-1387-x (PMC6417190; doi:10.1186/s13068-019-1387-x)
Supplement: Supplementary file 1 — Additional file 1: Fig. S1. Effects of severity factors (SFs, logR0) on the (A) average size and (B) zeta potential of microparticles in LHW-pretreated bamboo hydrolysates. Fig. S2. FTIR spectra of the raw bamboo material and LHW-pretreated bamboo substrates. Fig. S3. (A) XPS spectra and (B) Slig of the raw bamboo material and LHW–pretreated bamboo substrates. Fig. S4. SEM images of (A) untreated bamboo, and LHW-pretreated bamboo substrates labeled as (B) B-LHW-T180t20 (SF 3.66), (C) B-LHW-T180t40 (SF 3.96), (D) B-LHW-T200t15 (SF 4.12) and (E) B-LHW-T200t30 (SF 4.42). Fig. S5 Adsorption of cellulase (Celluclast 1.5 L®) and SP on the pretreated bamboo substrate (SF 4.42). Table S1. Mass balances of carbohydrates in the pretreatment liquors of bamboo. Table S2. ECs of glucan in different pretreated bamboo substrates at an enzymatic hydrolysis time of 96 h. Table S3. Remaining cellulase (Celluclast 1.5 L®) activities (FPU/mL) in the two bamboo enzymatic hydrolysates (SFs of 3.66 and 4.42) after 72 and 96 h hydrolysis at cellulase loadings of 24.2 and 96.7 mg protein/g glucan. Table S4. Concentrations of SP and glucose in the AA-SA solution (pH 5, 50 mmol/L) and the corresponding extraction ratio. Table S5. Zeta potentials, average sizes, and activities of cellulase, SP and cellulase-SP mixtures. Table S6. Cost estimation of SP as an additive during enzymatic hydrolysis. [file 13068_2019_1387_MOESM1_ESM.docx]

**Additional files**

**Promoting enzymatic hydrolysis of lignocellulosic biomass by inexpensive soy protein**

Xiaolin Luo^1^, Jing Liu^1^, Peitao Zheng^1^, Meng Li^1,2^, Yang Zhou^3^, Liulian Huang^1^, Lihui Chen^1*^, and Li Shuai^3*^

^1^ College of Materials Engineering, Fujian Agriculture and Forestry University, Fuzhou 350002, China

^2^ College of Energy, Xiamen University, Xiamen 361102, China

^3^ Department of Sustainable Biomaterials, Virginia Tech, 230 Cheatham Hall, Blacksburg, VA 24060, United States

*Corresponding authors.

Email: [lihuichen66@126.com](mailto:lihuichen66@126.com) (L. H. Chen)

Email: [shuai@vt.edu](mailto:shuai@vt.edu) (L. Shuai)

**Additional files**

**Contents:**

Additional experiment procedures (including references).

Fig. S1 Effects of severity factors (SFs, logR_0_) on the (A) average size and (B) zeta potential of microparticles in LHW-pretreated bamboo hydrolysates.

Fig. S2 FTIR spectra of the raw bamboo material and LHW-pretreated bamboo substrates.

Fig. S3 (A) XPS spectra and (B) S_lig_ of the raw bamboo material and LHW–pretreated bamboo substrates.

Fig. S4 SEM images of (A) untreated bamboo, and LHW-pretreated bamboo substrates labeled as (B) B-LHW-T180t20 (SF 3.66), (C) B-LHW-T180t40 (SF 3.96), (D) B-LHW-T200t15 (SF 4.12) and (E) B-LHW-T200t30 (SF 4.42).

Fig. S5 Adsorption of cellulase (Celluclast 1.5 L^®^) and SP on the pretreated bamboo substrate (SF 4.42).

Table S1 Mass balances of carbohydrates in the pretreatment liquors of bamboo.

Table S2 ECs of glucan in different pretreated bamboo substrates at an enzymatic hydrolysis time of 96 h.

Table S3 Remaining cellulase (Celluclast 1.5 L^®^) activities (FPU/mL) in the two bamboo enzymatic hydrolysates (SFs of 3.66 and 4.42) after 72 and 96 h hydrolysis at cellulase loadings of 24.2 and 96.7 mg protein/g glucan.

Table S4 Concentrations of SP and glucose in the AA-SA solution (pH 5, 50 mmol/L) and the corresponding extraction ratio.

Table S5 Zeta potentials, average sizes, and activities of cellulase, SP and cellulase-SP mixtures.

Table S6 Cost estimation of SP as an additive during enzymatic hydrolysis.

**Additional experiment procedures (including references)**

**Measurement of sugar and furanics concentrations in bamboo pretreatment hydrolysates**

The concentrations of monosaccharides, and furanics in bamboo pretreatment hydrolysates were measured by ion chromatography (IC, Dionex ICS-5000, US). The IC was equipped with a dual system. The system with an amperometric detector, a guard column, and an analytical column (Carbopac PA20) was used to measure carbohydrate concentrations at 30 °C; another system with a Supelcogel C-610H column and a UV detector was used to measure furanic concentrations at 210 nm and 20 °C. The detailed analysis procedures can be referred to a previous report [62].

The oligomeric saccharides (mainly xylo-oligosaccharides and gluco-oligosaccharides) in bamboo pretreatment hydrolysates were further hydrolyzed to monosaccharides by mixing the hydrolysates with an equal volume of sulfuric acid (8% (w/w)). The sulfuric acid hydrolysis was processed in an autoclave at 121 °C for 1 h. Total concentrations of monosaccharides in hydrolyzed pretreatment hydrolysates were also determined by IC with the same analysis procedure [62]. The difference between the total saccharide concentration and the monosaccharide concentration was calculated as the concentration of oligosaccharides in a hydrolysate.

**Measurement of colloid size and its zeta potential**

Average size and zeta potential of microparticles in pretreated bamboo hydrolysates were analyzed by a dynamic light scattering (DLS) analyzer equipped with a laser Doppler microelectrophoresis (Zetasizer Nano ZS90, Malvern Instruments, Malvern, UK). Before measurement, pretreatment hydrolysates were filtered by 0.45 μm syringe membranes (Millipore, Billerica, MA, USA). Deionized water was used for background correction.

Without the inputs of pretreated bamboo substrates, cellulase (Celluclast 1.5 L^®^), SP extract and their mixture diluted by an AA-SA buffer solution (pH 5.0, 50mmol/L) were also analyzed by DLS for average size and zeta potential measurements. For these tests, the AA-SA buffer solution was used for background correction.

**Lignin isolation**

According to the method reported by previous reports [65, 66], the lignin used in this research was isolated from the bamboo substrate labeled as B-LHW-T200t30 with an SF of 4.42. Initially, the substrate was freeze-dried and ground in a mill (ZM 200, Retsch, Haan, Germany) to pass a screen with a square opening size of 180 μm. The carbohydrates in the resulted powder of substrate were removed by extensive enzymatic hydrolysis. The enzymatic hydrolysis was conducted at 50 °C and 200 rpm for 48 h and repeated three times. The substrate, cellulase (Celluclast 1.5 L^®^), cellobiase (Novozyme 188) and xylanase (Pentopan Mono BG^®^, 2500U/g, Sigma-Aldrich, Shanghai, China) loadings for each time of enzymatic hydrolysis were 2% (w/v), 72.5 mg protein (30 FPU)/g glucan, 23.8 mg protein (45 CBU)/g glucan and 10 mg protein/g solid substrate (on o.d. basis), respectively. After the repeated enzymatic hydrolysis, the hydrolysate was filtered on a Büchner funnel with filter paper. Resulted crude lignin was further washed by deionized water (2%, w/w) and sodium phosphate buffer (50 mmol/L, pH 7.0, 2% (w/w)) at room temperature for 60 min, respectively. According to previous reports [66, 67], residual enzymes in washed lignin were further hydrolyzed by protease (2.4 U/mL, Sigma-Aldrich, Shanghai, China). This hydrolysis reaction was conducted with a protease loading of 0.2 mL/g lignin in a 50 mmol/L sodium phosphate buffer (2% lignin, w/v) at 50 °C and 200 rpm for 24 h. At the end of hydrolysis, the protease in hydrolysate was deactivated by heating the enzymatic hydrolysis hydrolysate at 90 °C for 30 min. Finally, the hydrolysate was filtered to collect the lignin which was further impregnated in NaCl solution (200 mL, 1 mol/L) and 200 mL deionized water at room temperature for 60 min, respectively. After centrifugation and freeze-drying, the purified lignin was obtained.

**SEM, FTIR and XPS characterization**

The surfaces of bamboo raw material and LHW pretreated bamboo substrates without grinding were imaged by field emission scanning electron microscopy (SEM, JEOL JSM-7500F, Japan). Vacuum dried samples were initially coated by the gold through vacuum sputtering and then subjected to SEM test under high vacuum operation mode at 3.0 kV.

Vacuum dried samples were further characterized by X-ray photoelectron spectroscopy (XPS, ESCALAB250, Thermo-Fisher Scientific, US). For XPS characterizations, the survey spectra were collected from 200 to 600 eV and used to determine the compositions of C and O elements on the surfaces of samples. As Laine et al. [68] described, the relative peak area of C and O elements on the surfaces of holocellulose (termed as carbohydrate) and lignin were different from each other, in which O/C_carbohydrate_ for former and O/C_lignin_ for later had been measured to be 0.83 and 0.33, respectively. Thus, an empirical function was proposed to quantify the surface lignin coverage (S_lig_, %) of the samples [68]. The calculation equation can be expressed as follows:

$S_{\mathrm{lig}}= 100\frac{{O/C}_{\mathrm{sample}} - {O/C}_{\mathrm{carbohydrate}}}{{O/C}_{\mathrm{lignin}} - {O/C}_{\mathrm{carbohydrate}}}$ (S1)

where O/C_sample_, O/C_carbohydrate,_ and O/C_lignin_ refer to the ratios of relative contents of O to C elements on the surfaces of the sample, holocellulose (0.83) and milled wood lignin (0.33), respectively.

Vacuum dried bamboo samples were finely ground using a mill (ZM 200, Retsch, Haan, Germany) to pass a screen with a square opening size of 180 μm. Resulted powders (1 wt %) were mixed with KBr for FTIR (Thermo-Nicolet AVATAR 380, USA) measurement. FTIR spectra were recorded in the range of 4000 ~ 400 cm^-1^ with a resolution of 4 cm^-1^.

**Measurement of cellulase activity**

The activities of cellulase preparations (Celluclast 1.5 L^®^ and Cellic CTec2) and cellulase-SP mixture in AA-SA buffer solutions, and cellulase in enzymatic hydrolysates were measured according to the IUPAC method [60]. Briefly, cellulase activity was measured by the filter paper method. First, 0.5 ml of the sample liquor was added to a test tube containing 1 mL of sodium citrate buffer (50 mmol/L, pH 4.8). Then, a Whatman No. 1 filter paper strip with a length and width of 6 cm × 1 cm was placed below the liquid level of the test tube. After sealing with a rubber stopper, the test tube was incubated in a water bath at 50 °C for 1 h. After the reaction, the concentration of reducing sugar in the liquid was measured by the dinitrosalicylic acid (DNS) method. Based on the above-mentioned IC method [62], the glucose concentration in the buffered cellulase preparations, the cellulase-SP mixture solutions, and the enzymatic hydrolysates were measured to be less than 0.5 g/L. Probably due to detection limits, no xylose and cellobiose were detected. The interferences of these background glucose concentrations on the DNS determination were initially subtracted. One filter paper unit (FPU) of enzyme activity is defined as a 1 μmoL concentration of reducing sugar produced per minute.

**Adsorption of cellulase and SP on the substrate**

The adsorption of cellulase (Celluclast 1.5 L^®^) and/or SP on a pretreated bamboo substrate (labeled as B-LHW-T200t30) was studied according to a previous report [65]. Cellulase and SP loadings were 24.2 mg protein (10 FPU)/g glucan and 160 mg protein/g glucan in the bamboo substrate, respectively. Other conditions were the same as that of the enzymatic hydrolysis. The liquor was sampled at 2, 6, 12, 24, 48 and 72 h, respectively. The hydrolysate samples were centrifuged (Avanti J-30I, Beckman Coulter Inc., Fullerton, CA, USA) at 8000 rpm for 2 min, and then filtered with a syringe filter (0.22 μm, Millex GV, Millipore). The concentrations of protein in the filtrate before and after adsorption were measured by bicinchoninic acid (BCA) protein assay kit (Thermo Scientific life, Rockford, IL, USA) [61]. The glucose concentrations in the received cellulase preparation (Celluclast 1.5 L^®^) and SP extract (Table S4) were measured to be 0.412 mg/mL and 0.075 mg/mL, respectively. The final measured background glucose concentrations in the AA-SA buffer solution based on the above dosages were both less than 0.1 mg/mL. According to the report from Mok et al. [69], we had previously determined the contribution of glucose concentration (C_glu_) to the absorbance at 562 nm ($A_{562}^{\mathrm{glu}}$) during the BCA test. According to Lambert Beer's law, the relationship between them was fitted as follows:

$A_{562}^{\mathrm{glu}}=\varepsilon_{562}^{\mathrm{glu}}C_{\mathrm{glu}}$ (S2)

where $\varepsilon_{562}^{\mathrm{glu}}$ is the molar absorption coefficient of glucose at 562 nm during the BCA test and fitted to be 0.078 mL/mg; the square of the correlation coefficient between the $A_{562}^{\mathrm{glu}}$ and the C_glu_ is 0.995.

Due to the additive principle of UV-Vis absorption [70], the total absorbance at 562 nm ($A_{562}^{T}$) is the sum of protein ($A_{562}^{\mathrm{pro}}$) and glucose ($A_{562}^{\mathrm{glu}}$). This relationship can be presented as:

$A_{562}^{T}=A_{562}^{\mathrm{glu}}+A_{562}^{\mathrm{pro}}$ (S3)

When BSA was used as a protein standard, the molar absorption coefficient of SP or cellulase at 562 nm ($\varepsilon_{562}^{\mathrm{pro}}$) was fitted to be 0.792 mL/mg (R^2^ = 0.996). Therefore, the concentration of proteins (C_pro_, mg/mL) in the buffer solution determined by BCA method can be calculated as:

$C_{\mathrm{pro}}={(A}_{562}^{T}-0.078C_{\mathrm{glu}})/0.792$ (S4)

For the data reported in this article, the interference of glucose on protein concentrations during the BCA test had been ruled out with these operations.

**References (65 – 70)**

65. Ko JK, Ximenes E, Kim Y, Ladisch MR. Adsorption of enzyme onto lignins of liquid hot water pretreated hardwoods. Biotechnol Bioeng. 2015;112:447–56.

66. Kumar R, Wyman CE. Access of cellulase to cellulose and lignin for poplar solids produced by leading pretreatment technologies. Biotechnol Prog. 2009;25:807-19.

67. Yang B, Willies DM, Wyman CE. Changes in the enzymatic hydrolysis rate of Avicel cellulose with conversion. Biotechnol Bioeng. 2006;94:1122–28.

68. Laine J, Stenius P, Carlsson G, Ström G. Surface characterization of unbleached kraft pulps by means of ESCA. Cellulose 1994;1:145–60.

69. Mok YK, Arantes V, Saddler JN. A NaBH₄ coupled ninhydrin-based assay for the quantification of protein/enzymes during the enzymatic hydrolysis of pretreated lignocellulosic biomass. Appl Biochem Biotechnol. 2015;176:1564-80.

70. Luo XL, Zhan HY, Chai XS, Fu SY, Liu J. A novel method for determination of aromatic aldehyde monomers in lignin degradation liquor. Ind Eng Chem Res. 2009;48:2713-16.

**Fig. S1**

Fig. S1 Effects of severity factors (SFs, logR_0_) on the (A) average size and (B) zeta potential of microparticles in LHW-pretreated bamboo hydrolysates.

**Fig. S2**

Fig. S2 FTIR spectra of the bamboo raw material and LHW-pretreated bamboo substrates.. The substrates were pretreated by LHW pretreatment with SFs ranged from 3.66 to 4.42.

**Fig. S3**

Fig. S3 (A) XPS spectra and (B) S_lig_ of the bamboo raw material and LHW–pretreated bamboo substrates.

**Fig. S4**


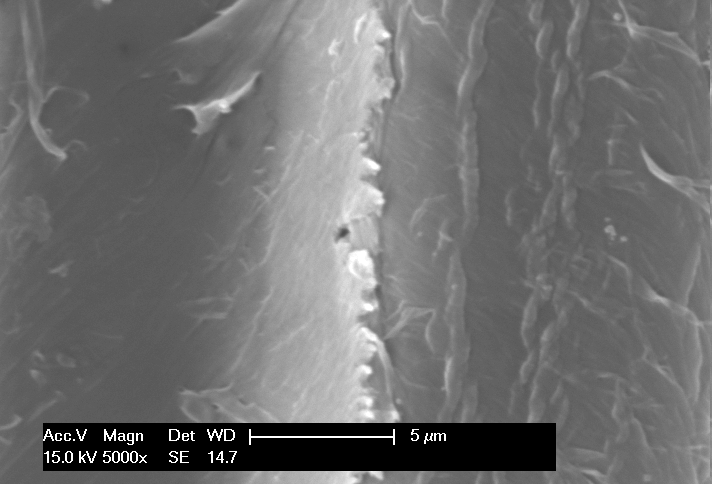

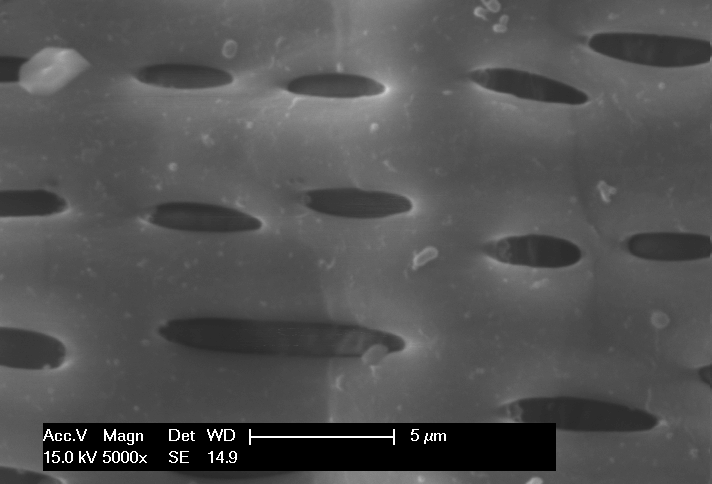


**B**

**A**


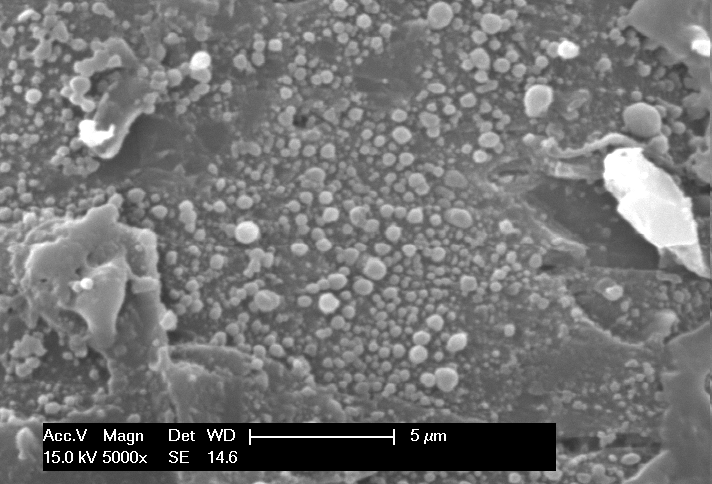

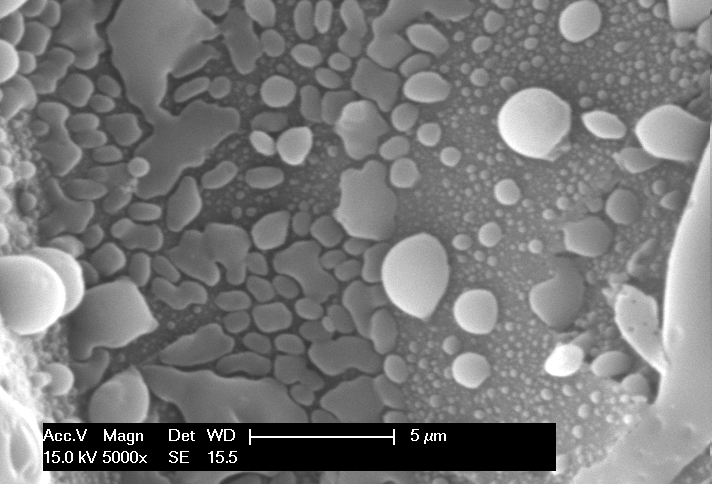

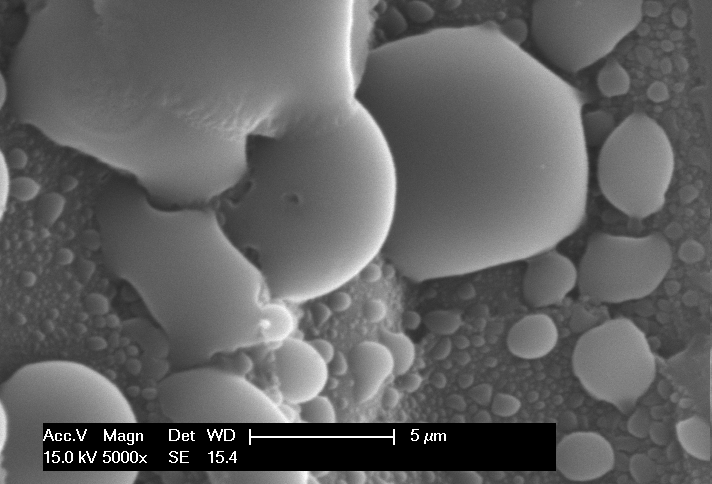


**E**

**D**

**C**

Fig. S4 SEM images of (A) untreated bamboo, and LHW-pretreated bamboo substrates labeled as (B) B-LHW-T180t20 (SF 3.66), (C) B-LHW-T180t40 (SF 3.96), (D) B-LHW-T200t15 (SF 4.12) and (E) B-LHW-T200t30 (SF 4.42).

B and LHW refer to the abbreviations of bamboo and liquid hot water; the numbers after T and t stand for the pretreatment temperature (°C) and duration (min), respectively.

**Fig. S5**

**Fig. S5** Adsorption of cellulase (Celluclast 1.5 L^®^) and SP on the pretreated bamboo substrate (SF 4.42). (A) The concentration of residual protein in buffer solution and (B) the amount of adsorbed protein on the pretreated substrate after an adsorption time of 72 h. The substrate loading was 2 g/100 mL AA-SA buffer solution.

**Table S1**

**Table S1** Mass balances of carbohydrates in pretreatment hydrolysates of bamboo. The mass balances were conducted based on the concentration of cellulose and hemicellulose-derived oligo-sugars, mono-sugars and furanics in pretreatment hydrolysates.

| Label | Temp.  (°C) | Time (min) | SF (logR_0_) | X (g/L) | XO  (g/L) | F  (g/L) | TXDSC (g/L) | XR  (%) | G  (g/L) | GO  (g/L) | HMF  ( g/L) | TGDSC (g/L) | GR  ( %) |
| --- | --- | --- | --- | --- | --- | --- | --- | --- | --- | --- | --- | --- | --- |
| B-LHW-T160t20 | 160 | 20 | 3.07 | 0.08 | 1.99 | 0.05 | 1.93 | 83.00 | 0.11 | 1.60 | 0.00 | 1.54 | 92.12 |
| B-LHW-T160t40 | 160 | 40 | 3.37 | 0.14 | 3.57 | 0.09 | 3.47 | 78.62 | 0.22 | 3.54 | 0.00 | 3.38 | 91.43 |
| B-LHW-T180t20 | 180 | 20 | 3.66 | 0.58 | 7.58 | 0.23 | 7.67 | 77.83 | 0.92 | 5.81 | 0.02 | 6.08 | 89.26 |
| B-LHW-T180t40 | 180 | 40 | 3.96 | 2.29 | 13.88 | 0.56 | 15.34 | 74.63 | 1.19 | 6.09 | 0.09 | 6.66 | 87.89 |
| B-LHW-T200t15 | 200 | 15 | 4.12 | 4.14 | 13.16 | 1.51 | 17.69 | 68.61 | 2.20 | 6.97 | 0.16 | 8.45 | 85.33 |
| B-LHW-T200t30 | 200 | 30 | 4.42 | 10.69 | 5.13 | 5.49 | 21.96 | 65.06 | 4.32 | 3.43 | 0.74 | 7.93 | 83.89 |

P, X, XO, F, TXDSC, XR, G, GO, HMF, TGDSC, GR stand for P-factor, xylose, xylo-oligosaccharides, furfural, total xylan-derived sugar concentration, xylan recovery, glucose, gluco-oligosaccharides, 5-hydroxymethylfurfural, total glucan-derived sugar concentration, glucan recovery, respectively.

Xylan and glucan recovery were calculated on the basis of corresponding sugars removed from raw material into pretreatment hydrolysate.

TXDSC (g/L) = X*0.9 + XO*0.9 + F*0.9*150/96 (S5)

TGDSC (g/L) = G*0.9 + GO*0.9 + HMF*0.9*180/126 (S6)

where 0.9 is the conversion coefficient of glucose to glucan during enzymatic hydrolysis.

For simplifying the calculations of XR and GR, we assume that F and HMF only degraded from xylose and glucose, respectively.

$XR=\frac{TXDSC\times\eta\times W_{RM}\div1000}{W_{RM}\times C_{X}\times R_{X}\div100}$ (S7)

where *XR* is the xylan recovery (%); *ƞ* is the ratio of deionized water to chips (on o.d. basis) (6:1, mL: g); *W_RW_* is the weight (g) of raw material used for LHW pretreatment; *C_X_* is the content (%) of xylan in raw material; *R_X_* is the xylan removal (%) after conducted LHW pretreatment, respectively.

$GR=\frac{TGDSC\times\eta\times W_{RM}\div1000}{W_{RM}\times C_{G}\times R_{G}\div100}$ (S8)

where *GR* is the glucan recovery (%); *C_G_* is the content (%) of glucan in raw material; *R_G_* is the glucan removal (%) after conducted LHW pretreatment, respectively.

**Table S2**

**Table S2** ECs of glucan in different pretreated bamboo substrates with an enzymatic hydrolysis time of 96 h.

| Label | SF (logR_0_) | Celluclast 1.5 L^®^ loading (mg protein/g glucan) | | | | |
| --- | --- | --- | --- | --- | --- | --- |
|  |  | 6.0 | 12.1 | 24.2 | 48.3 | 96.7 |
| B-LHW-T160t20 | 3.07 | 3.45 | 3.96 | 4.43 | 4.97 | 5.66 |
| B-LHW-T160t40 | 3.37 | 4.78 | 5.26 | 6.22 | 7.57 | 8.44 |
| B-LHW-T180t20 | 3.66 | 6.57 | 8.61 | 11.46 | 14.16 | 16.73 |
| B-LHW-T180t40 | 3.96 | 12.37 | 18.42 | 27.45 | 36.18 | 44.69 |
| B-LHW-T200t15 | 4.12 | 14.77 | 22.16 | 31.74 | 40.21 | 49.81 |
| B-LHW-T200t30 | 4.42 | 27.92 | 40.00 | 57.94 | 82.82 | 93.41 |

**Table S3**

**Table S3** Remaining cellulase (Celluclast 1.5 L^®^) activities (FPU/mL) in two bamboo enzymatic hydrolysates (SFs of 3.66 and 4.42) after 72 and 96 h hydrolysis with cellulase loadings of 24.2 and 96.7 mg protein/g glucan.

| Enzymatic hydrolysis duration (h) | Celluclast 1.5 L^®^ loading (mg protein/g glucan)^a^ | Enzymatic hydrolysates^b^ | |
| --- | --- | --- | --- |
|  |  | B-LHW-T180t20 | B-LHW-T200t30 |
| 72 | 24.2 | 0.014/17% | 0.003/3% |
|  | 96.7 | 0.117/35% | 0.090/22% |
| 96 | 24.2 | 0.010/12% | 0.002/2% |
|  | 96.7 | 0.090/27% | 0.073/18% |

^a^ The activity of used cellulase is 51.4 FPU/mL. No cellobiase (Novozyme 188) was added during this process.

^b^ The numbers after slash are the percentage of remain cellulase activity in the enzymatic hydrolysate. The percentage of remain cellulase activity is equal to the activity of cellulase in enzymatic hydrolysate after 72 and 96 h hydrolysis divided by the activity of cellulase diluted in corresponding buffer solution.

**Table S4**

**Table S4** Concentration of SP and sugar in buffered extraction solution (pH 5, 50mmol/L) and corresponding extraction ratio.

|  | SP | Glucose | Xylose |
| --- | --- | --- | --- |
| Concentration (g/L) | 13.200 | 0.075 | 0.000 |
| Extraction ratio (%, w/w) | 6.60 | 0.04 | 0.00 |

**Table S5**

**Table S5** Zeta potentials, average sizes, and activities of cellulase, SP and cellulase-SP mixtures.

|  | Cellulase | SP | Cellulase-SP mixture |
| --- | --- | --- | --- |
| Zeta potential^a^ (mV) | -0.7 ± 0.2 | -7.9 ± 1.6 | -8.4 ± 1.1 |
| Average size^a^ (nm) | 11.8 ± 1.9 | 515.7 ± 115.6 | 391.6 ± 40.9 |
| Activity^b^ (FPU/mL) | 48.6 ± 1.5 | N.D.^d^ | 48.4 ± 1.3 |
| Activity^c^ (FPU/mL) | 30.3 ± 0.9 | N.D.^d^ | 35.3 ± 1.1 |

^a^ Cellulase (Celluclast 1.5 L^®^) and SP extract (Table S4) were diluted 1000 and 100 times with AA-SA buffer (pH 5.0, 50mmol/L) for zeta potential and average size measurements, respectively. Diluted cellulase and SP were further mixed at a volume ratio of 1:1 to conduct corresponding measurements.

^b^ For the ease of comparison, the activities of cellulase were calculated based on the dilution times. The volume of cellulase-SP mixture or cellulase alone was 10 mL, and it was incubated in a *25-mL Erlenmeyer flask* at 150 rpm and 50 °C for 72 h.

^c^ The mixture or cellulase alone was 10 mL and incubated in a *125-mL Erlenmeyer* flask at same conditions.

^d^ N.D. presents “not detected”.

It was found that the surface net charge of cellulase (Celluclast 1.5 L^®^) and SP were both negative in the AA-SA buffer with a pH of 5 (Table 3). After mixing at 50 °C for 72 h, the average size of the cellulase-SP mixture did not increase, indicating that there was essentially no interaction between them.

**Deactivation of enzymes at the air-liquid interface [25]**

With a high interfacial area of enzymatic hydrolysate (10 mL liquor in 25 mL reactor volume), cellulase activity was not affected by SP and/or SP could somehow help cellulase to avoid the loss of enzyme activity. However, with a low interfacial area of enzymatic hydrolysate (10 mL liquor in 125 mL reactor volume), cellulase lost some activity and SP could help to reduce the loss of enzyme activity. However, the interactions of cellulase and SP could be very complicated and need additional investigations.

**Table S6**

**Table S6** Cost estimation of SP as an additive during enzymatic hydrolysis. The enzymatic hydrolysis (EH) was referred to the pretreated bamboo substrate labeled as B-LHW-T200t30.

|  | Celluclast 1.5 L^®^ only | Celluclast 1.5 L^®^ + SP | |
| --- | --- | --- | --- |
|  |  | Celluclast 1.5 L^®^ | SP |
| Celluclast 1.5 L^®^ loading (FPU/g glucan) | 10 | 10 |  |
| Protein charge (mg/g glucan) or (mg/g substrate) ^a^ | 24.2/12.6 | 24.2/12.6 | 160/83.4 |
| ECs of glucan in the substrate (%) | 57.9 | 97.6 | |
| Adsorbed protein after one time of EH (mg/g) substrate) ^b^ | 6.2 | 28.0 | |
| Supplemented protein for subsequent 19 EHs (mg/g glucan) | 24.2 | 24.2 | 3.8 |
| The price of cellulase or commercial SP ($/kg protein) ^c^ | 5.07 | 5.07 | 1.25 |
| Equivalent cellulase loading for each EH (mg protein or FPU/g glucan) ^d^ | 24.2/10 | 27.1/11.2 | |
| Final glucose concentration after 20 times of EH (g/L) ^d^ | 132.7 | 223.7 | |

^a^ The glucan content of the bamboo substrate labeled as B-LHW-T200t30 is 52.1% (w/w, on the basis of the o.d. weight of the substrate).

^b^ The adsorption was continued for 72 h, which could be referred from Fig. S5.

^c^ The price of cellulase or commercial SP ($/kg) were cited from the references [12]. Herein, the lowest price of commercial cellulase was used. If a higher cellulase price ($ 6.27 – 23.30 kg/protein) was adopted for the calculation, the ratio of SP cost to enzyme cost will be even lower.

^d^ The detailed calculations of SP cost during high-consistency enzymatic hydrolysis was shown below.

The protein content and enzyme activity of cellulase (Celluclast 1.5 L^®^) are 124.2 mg protein/mL and 51.4 FPU/ml, respectively. For a cellulase loading of 10 FPU/g glucan in the bamboo substrate, the protein charge can be calculated as: 124.2 (mg protein/mL)* [10 (FPU/g glucan)/51.4 (FPU/mL)] = 24.2 (mg protein/g glucan).

After 20 cycles of feeding the fresh substrate and enzymatic hydrolysis, the total loading of SP can be calculated as: 160 (mg/g glucan) + 3.8 (mg/g glucan) × 19 times = 232.2 (mg/g glucan in bamboo substrate). The average loading of SP for each cycle is: 232.2 (mg/g glucan)/20 times = 11.61 (mg/g glucan). The unit price of cellulase and SP are US $5.07 and $1.25/kg protein, respectively. Based on the their prices, the equivalent cellulase loading of the loaded SP for each cycle is: 11.61 (mg/g glucan) × [($1.25/kg protein) / ($5.07/kg protein)] / [ 24.2 (mg protein/g glucan)/ 10 (FPU/g glucan)] = 1.2 (FPU/g glucan).
